# Emotions and Topics Expressed on Twitter During the COVID-19 Pandemic in the United Kingdom: Comparative Geolocation and Text Mining Analysis

**DOI:** 10.2196/40323

**Published:** 2022-10-05

**Authors:** Hassan Alhuzali, Tianlin Zhang, Sophia Ananiadou

**Affiliations:** 1 College of Computers and Information Systems Umm Al-Qura University Makkah Saudi Arabia; 2 Department of Computer Science National Centre for Text Mining The University of Manchester Manchester United Kingdom; 3 The Alan Turing Institute London United Kingdom

**Keywords:** Twitter, COVID-19, geolocation, emotion detection, sentiment analysis, topic modeling, social media, natural language processing, deep learning

## Abstract

**Background:**

In recent years, the COVID-19 pandemic has brought great changes to public health, society, and the economy. Social media provide a platform for people to discuss health concerns, living conditions, and policies during the epidemic, allowing policymakers to use this content to analyze the public emotions and attitudes for decision-making.

**Objective:**

The aim of this study was to use deep learning–based methods to understand public emotions on topics related to the COVID-19 pandemic in the United Kingdom through a comparative geolocation and text mining analysis on Twitter.

**Methods:**

Over 500,000 tweets related to COVID-19 from 48 different cities in the United Kingdom were extracted, with the data covering the period of the last 2 years (from February 2020 to November 2021). We leveraged three advanced deep learning–based models for topic modeling to geospatially analyze the sentiment, emotion, and topics of tweets in the United Kingdom: SenticNet 6 for sentiment analysis, SpanEmo for emotion recognition, and combined topic modeling (CTM).

**Results:**

We observed a significant change in the number of tweets as the epidemiological situation and vaccination situation shifted over the 2 years. There was a sharp increase in the number of tweets from January 2020 to February 2020 due to the outbreak of COVID-19 in the United Kingdom. Then, the number of tweets gradually declined as of February 2020. Moreover, with identification of the COVID-19 Omicron variant in the United Kingdom in November 2021, the number of tweets grew again. Our findings reveal people’s attitudes and emotions toward topics related to COVID-19. For sentiment, approximately 60% of tweets were positive, 20% were neutral, and 20% were negative. For emotion, people tended to express highly positive emotions in the beginning of 2020, while expressing highly negative emotions over time toward the end of 2021. The topics also changed during the pandemic.

**Conclusions:**

Through large-scale text mining of Twitter, our study found meaningful differences in public emotions and topics regarding the COVID-19 pandemic among different UK cities. Furthermore, efficient location-based and time-based comparative analysis can be used to track people’s thoughts and feelings, and to understand their behaviors. Based on our analysis, positive attitudes were common during the pandemic; optimism and anticipation were the dominant emotions. With the outbreak and epidemiological change, the government developed control measures and vaccination policies, and the topics also shifted over time. Overall, the proportion and expressions of emojis, sentiments, emotions, and topics varied geographically and temporally. Therefore, our approach of exploring public emotions and topics on the pandemic from Twitter can potentially lead to informing how public policies are received in a particular geographical area.

## Introduction

The crisis of the COVID-19 pandemic has influenced the whole world on an enormous scale, causing most countries to deal with an unprecedented situation. The societal consequences due to lockdowns were tremendous on all levels. The pandemic caused most countries to impose various stages of restrictions on moving, traveling, and gathering to contain the outbreak of infection. Such restrictions changed how people used to work, socialize, shop, travel, etc, leading to various behavioral and societal changes to deal with the situation (eg, working from home, fear of social interaction, isolation, loneliness). Because of this unprecedented societal change, it was important for policymakers to understand people’s state of mind to help institutions, governments, and individuals navigate through the pandemic [1-4].

Traditionally, policymakers used questionnaires to capture public opinion toward major events, but there are disadvantages limiting the effectiveness of such methods of evidence gathering due to bias caused by spatiotemporal granularity and sample sizes. Recently, social media have become an important vehicle of gathering information and evidence about public opinion. Twitter is a popular social media platform with more than 19 million users in the United Kingdom [5], where there are many discussions and opinions about topics related to COVID-19. Previous studies show that Twitter can yield important public health information and has broad applicability for public health research, including medical well-being and tracking infectious disease outbreaks [6,7]. Therefore, to address the evidence gap from traditional surveys, Twitter data can be used to supplement data gathering, and to understand public opinion on pandemics [8,9] and reactions to the COVID-19 outbreak [10].

There is a growing body of research that has recently focused on the COVID-19 pandemic with respect to different attributes, including sentiment, emotions, and topics [11-16]. Kleinberg et al [11] built the COVID-19 Real World Worry Dataset, which is based on a direct survey written by 2500 participants who reported their feelings while writing. Gupta et al [13] created another COVID-19 data set from Twitter by using a set of keywords related to the pandemic, as well as analyzing sentiment and topics as additional attributes to emotion. For instance, there are some analyses of COVID-19 vaccine–related discussions on Twitter or Reddit based on sentiment analysis and topic modeling in different countries, including the United States [17-19], Canada [20], the United Kingdom [18], Saudi Arabia [21], and Australia [22].

Sentiment represents the attitude and feelings expressed by people. Sentiment analysis determines and interprets whether online posts collected from social media are positive, neutral, or negative, and helps to gain better insight into public perceptions and attitudes. Sentiment analysis can also help to understand how information spreads on social media: a tweet with positive/negative sentiment generates another tweet with the same or opposing sentiment [23]. Sentiment analysis has been used for many practical applications, including financial analysis, politics, health prediction, and health care service improvement [24]. For instance, by analyzing public messages, sentiment analysis can be used by health practitioners to understand potential obstacles to population-based intervention approaches such as COVID-19 vaccination. In addition, analyzing patients’ online reviews of different treatments can improve patient satisfaction [25].

Emotion detection from social media plays an important role in monitoring health and well-being [26]. Clinicians and health professionals also benefit from emotion analysis to understand public emotions and public health changes in perception of an intervention (ie, vaccine). Emotion detection systems have been used for alerting public health practitioners, for monitoring mental health patients [27], suicide prevention [28], and adverse drug reactions [29]. Some works utilized emotion-based features to specifically detect adverse drug reactions reported by users on social media, which can guide health professionals and pharmaceutical companies in making medications safer and advocating for patient safety [30-32]. Moreover, the idea of emotional contagion can further play a crucial role in either improving the overall well-being of users or preventing them from developing mental health problems. Kramer et al [33] stated that emotions can be transferred to others through *emotional contagion.* Emotional contagion makes people experience similar emotions, even if they are not aware of their emotional changes. On the one hand, other works found a strong link between people’s mental health problems (ie, depression and anxiety) and the outbreak of COVID-19 due to the intense exposure to negative content on social media [34,35]. On the other hand, one can also expose people to positive or desired emotions (eg, calm, joy, optimism, and rest) to improve their overall well-being [33].

Besides sentiment analysis and emotion detection, topic modeling is an important text analysis technology by grouping texts into different themes. Most models can find hidden topics without supervision, and therefore do not require training on specific data with predefined topics, which makes this approach suitable for analyzing social media data to determine what people are talking about on these platforms. Topic modeling has been used for many health applications during the COVID-19 pandemic [36], such as monitoring people’s concerns, predicting COVID-19 cases, and analyzing government responses. Topic modeling has played a crucial role in health information surveillance and public opinion monitoring [37].

Given the growing interest of research in understanding people’s opinions and emotions regarding the pandemic [37], the objective of this study was to use deep learning–based methods to understand public emotions on topics related to the COVID-19 pandemic in the United Kingdom through a comparative geolocation and text mining analysis on Twitter. Specifically, we utilized three advanced deep learning–based methods (ie, SenticNet [38], SpanEmo [39], and combined topic modeling [CTM] [40]), and then performed our analysis on a data set collected from Twitter to explore people’s sentiment, emotions, and topics toward COVID-19. We further included analyses of these attributes focused on understanding the impact of the pandemic over time. The overall goal of this study was to automatically capture the impact COVID-19 had on the UK population using emotion detection, sentiment analysis, and topic modeling.

## Methods

### Data Source

To develop our corpus, we used the Twitter application programming interface by collecting data via the use of several bounding boxes over multiple cities in the United Kingdom. We further used a list of keywords that are of relevance to the pandemic (eg, coronavirus, sars19, covid19, and NHS [National Health Service]). The data covered the period of the last 2 years (ie, 2020 and 2021). To acquire location labels on the data, we used the Python geocoding library “geopy” [41], which helps locate the coordinates of addresses (eg, Oxford Rd, Manchester M13 9PL), cities (eg, Manchester), countries (eg, United Kingdom), and landmarks (in the form of latitude and longitude coordinates) based on third-party geocoders and several other data sources. More specifically, we use “Nominatim” [42] as a third-party tool. As a result, we acquired a total of 516,427 tweets from 48 cities in this study.

The number of tweets per city and emoji is shown in Table 1 and Multimedia Appendix 1, respectively. We further highlight the 9 cities that were used for our analysis: Birmingham, Bristol, Leeds, Leicester, Liverpool, London, Manchester, Nottingham, and Sheffield. It is worth mentioning that these 9 cities are also among the top populated cities in the United Kingdom [43]. This shows that there is a link between the population size and the number of posted tweets from a given geolocation area. Multimedia Appendix 1 displays the top 50 tweets (according to percentage) associated with each individual emoji and its meaning, highlighting the usage of emojis expressing different health issues (eg, virus, face with medical mask, syringe, or vaccine) and mental health conditions (eg, hands pressed together).

**Table 1:** Number of tweets per city in the United Kingdom.

City	Tweets, n	Population, n
Bath	1698	105,730
Birmingham^a^	21,120	1,159,888
Blackburn	1092	121,475
Bradford	4980	368,485
Brighton	10,092	245,504
Bristol^a^	10,338	580,199
Cambridge	6894	149,155
Canterbury	2292	64,495
Carlisle	1098	74,536
Chelmsford	3894	119,468
Chester	3516	87,881
Chichester	864	31,881
Coventry	6072	388,793
Derby	3503	264,430
Durham	9414	56,920
Ealing	4914	340,341
Ely	432	20,333
Exeter	3360	127,709
Gloucester	1740	148,167
Hereford	1134	64,037
Kingston	5286	287,705
Kirklees	3156	441,290
Lancaster	876	52,935
Leeds^a^	11,628	516,298
Leicester^a^	19,818	472,897
Lichfield	792	34,686
Lincoln	4614	107,434
Liverpool^a^	15,876	589,774
London^a^	111,667	9,088,994
Luton	2658	222,043
Manchester^a^	25,260	567,334
Newcastle	9642	290,688
Northampton	3954	230,070
Norwich	4290	199,245
Nottingham^a^	11,827	320,536
Peterborough	2054	179,349
Plymouth	2736	240,297
Portsmouth	4878	248,748
Preston	3816	100,095
Redbridge	3227	310,330
Ripon	138	15,971
Rochdale	1415	114,511
Rotherham	198	111,158
Salford	8034	125,983
Sheffield^a^	15,582	557,039
Southampton	7806	270,333
Worcester	3492	101,816
York	5748	164,934

^a^Top nine cities used in subsequent analyses.

### Methodology

**Figure 1:**
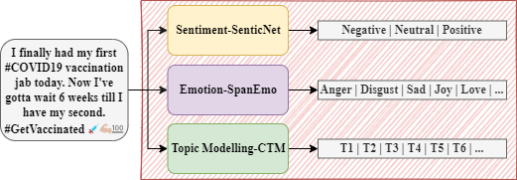
Overview of our pipeline. CTM: combined topic modeling.

To preprocess the data, we used the “ekphrasis” tool designed for the specific characteristics of Twitter (ie, misspellings and abbreviations) [44]. The tool provides different functionalities such as tokenization, normalization, and spelling correction. We utilized the tool to tokenize the text; convert words to lowercase; and normalize user mentions, URLs, and repeated characters. Once the preprocessing step was complete, we fed the data through three models: (1) a textual emotion deep learning–based recognition model, (2) a deep learning–based sentiment model, and (3) a neural network topic model. Figure 1 depicts our pipeline, in which we provide an illustration of the three deep-learning models.

We used SenticNet 6 [38] for sentiment analysis, since this model has achieved better performance than other machine learning–based sentiment analysis methods. SenticNet 6 can provide sentiment scores (between –1 and 1) for approximately 200,000 common-sense concepts by using both symbolic models (ie, logic and semantic networks) and subsymbolic methods with deep learning architectures to encode the meanings and syntactic relations. We then added up the sentiment scores of each concept in the post and used two basic linguistic patterns (negation and adversative patterns) [45]. For example, if the patterns are not used, “The television is old but rather not expensive” could be wrongly classified although both “old” and “expensive” are negative. Finally, we calculated the sentiment polarity of each post automatically. We divided our data into five categories based on the following score range: strong negative (–1 to –0.5), weak negative (–0.5 to –0.1), neutral (–0.1 to 0.1), weak positive (0.1 to 0.5), and strong positive (0.5 to 1).

The emotion recognition model is based on our deep learning–based model “SpanEmo” [39] that is designed for multilabel emotion classification. This model is specifically trained on the SemEval-2018 multilabel emotion classification data set [46], labeled with multiple emotions classes (ie, anger, anticipation, disgust, fear, joy, love, optimism, pessimism, sadness, surprise, and trust). SpanEmo focuses on both learning emotion-specific associations and integrating their correlations into the training objective. Since SpanEmo achieved strong performance for the task of multilabel emotion classification, we decided to use it to generate predictions for our data. It should be mentioned that only examples with high predictions are retained.

Last, for topic modeling, we used CTM [40]. This model incorporates contextualized document embeddings into neural topic models to produce more coherent and meaningful topics. Because the evaluation results on five publicly available data sets illustrate that the performance achieved by CTM is better than traditional latent Dirichlet allocation [47] topic models and other neural models, we employed CTM to extract the topics and their associated words from our data.

### Ethical Considerations

Since our data were collected from Twitter, we followed Twitter’s terms of service and strict ethical research protocols similar to the guidelines [48], protecting the privacy and security of personal data. It should be mentioned that our study was focused on the tweet level; we do not anticipate any negative ethical impact from our analysis. However, we believe that these results provide insights into people’s emotions and topics among different cities in the United Kingdom during the COVID-19 pandemic.

## Results

### Words Associated With Emotions

We performed different types of analyses focused on sentiment, emotion, and topic modeling of the COVID-19 online data sets. First, we analyzed emotion-words and topic-words associations where both demonstrate the relationship between words and their respective emotion label and topic. We then analyzed where the location is given, and where the impact of COVID-19 on different cities in the United Kingdom is discussed. Furthermore, an analysis of time-based features was undertaken, focusing on showing the impact of COVID-19 over time. Finally, we analyzed instances from our data that discuss the benefits of considering sentiment, emotion, and topical analysis in understanding the concerns of people during the pandemic in the United Kingdom.

Table 2 presents the top 6 words associated with each emotion and learned by SpanEmo. More detailed information on how to generate these words is provided by Alhuzali and Ananiadou [39]. There were words that are indicative of both the corresponding emotion as well as the COVID-19 pandemic. For instance, the words “death” and “spread” were highly associated with the emotion class fear, whereas words such as “vaccine” and “support” were highly associated with the emotion class anticipation. This is intuitive since some words directly express emotion (eg, angry, afraid, and glad), while other words indirectly express emotion (eg, accident, failure, and birthday). We also observed that some emotion classes shared similar words, especially those that belong to the same valence space [49]. The analysis presented in Table 2 demonstrates that it is possible to understand the impact of COVID-19 with the help of emotion analysis and the concerns of people during the pandemic.

We extracted topics using CTM. Table 3 summarizes the top 18 topics extracted as well as the top 5 associated words per topic. We noticed that there were many different topics mentioned by users, ranging from those related to COVID-19, such as epidemic control, government policies, and vaccination, to indirectly related subjects such as work, online, and social networking. For example, topic 1 (t1) contains some words about gratitude (ie, *grateful, thank)*, which is related to the attitude toward social support and vaccination. Topic 3 (t3) is about the discussion during the pandemic, topic 10 (t10) centers on the serious consequences of COVID-19 (*die, killing*), and topic 8 (t8) reveals occupational patterns.

**Table 2:** The top 6 words associated with each emotion class, predicted by SpanEmo.

Emotion class			Associated words
**Negative emotions**			
	Anger	death, think, public, virus, don’t, against	
	Disgust	deaths, virus, against, because, public, after	
	Fear	deaths, spread, symptoms, coronavirus, identify, self-reporting	
	Sadness	deaths, going, cases, hospital, other, please	
	Pessimism	sadly, family, friend, during, weeks, passed	
**Positive emotions**			
	Anticipation	support, vaccine, first, working, public, cases	
	Joy	great, thank, support, happy, amazing, staysafe	
	Trust	trust, thank, protect, important, community, everyone	
	Love	happy, loved, share, beautiful, wonderful, amazing	
	Optimism	please, thank, support, working, great, spread	
	Surprise	shocking, surprised, amazing, public, absolutely, deaths	

**Table 3:** Topics extracted using combination topic modeling and the top 5 associated words per topic.

Topic	Associated words
t1	thank, grateful, proud, amazing, heroes
t2	class, sign, trade, worldwide, hold
t3	discuss, blog, discussion, recovery, opportunities
t4	united, fitness, kingdom, complete, image
t5	episode, tune, film, videos, radio
t6	rear, accord, whack, discomfort, fills
t7	vaccination, vaccine, dose, drug, booster
t8	letter, homes, worker, pay, private
t9	visit, eye, tweet, click, website
t10	die, dying, true, killing, cause
t11	confirmed, total, English, wales, reports
t12	rear, accord, jeopardise, unknowingly, discomfort
t13	lies, cummings, press, leader, prime
t14	coronavirus, pandemic, outbreak, instagram, outbreak
t15	masks, wear, face, hand, covering
t16	slow, thread, implement, testandtrace, symptom
t17	couple, havent, felt, daughter, holiday
t18	stay, loved, tough, pray, healthy

### Analysis of Location

**Figure 2:**
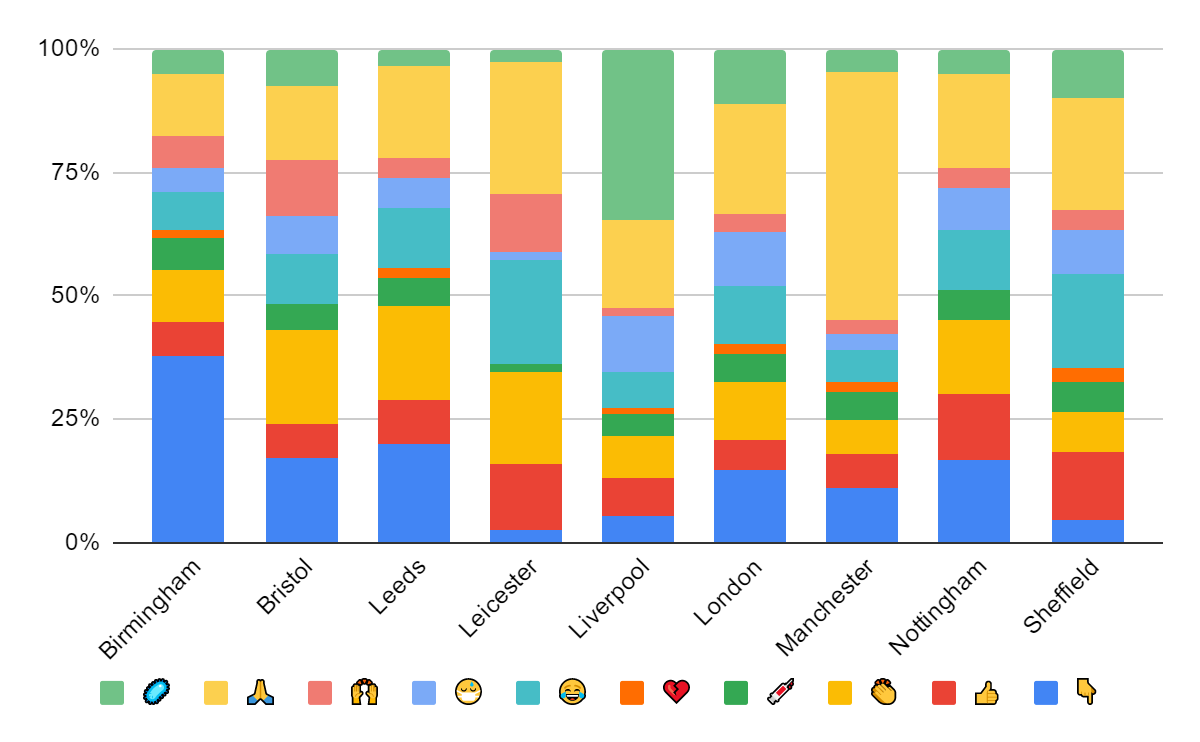
The number of emojis used across a sample of UK cities.

Figure 2 shows the number of emojis across a sample of UK cities, where the sample consists of the top 9 cities in our data, more specifically those that had the highest number of tweets (Table 1): Bristol, Birmingham, Leicester, Leeds, Liverpool, London, Manchester, Nottingham, and Sheffield. The emoji set included the following topics: virus, face-mask, thumbs-up/-down, broken heart, and others. The proportion of emojis differed from city to city. For example, usage of the syringe, or known today as the COVID-19 vaccine emoji, was high in Liverpool; the thumbs-down emoji was high in Birmingham; and the mask emoji was highly used in London and Liverpool. These emojis are relevant to the COVID-19 pandemic, demonstrating the benefits of our data in mining and analyzing social data such as Twitter for a better understanding of the impact of the pandemic on people from different areas in the United Kingdom.

**Figure 3:**
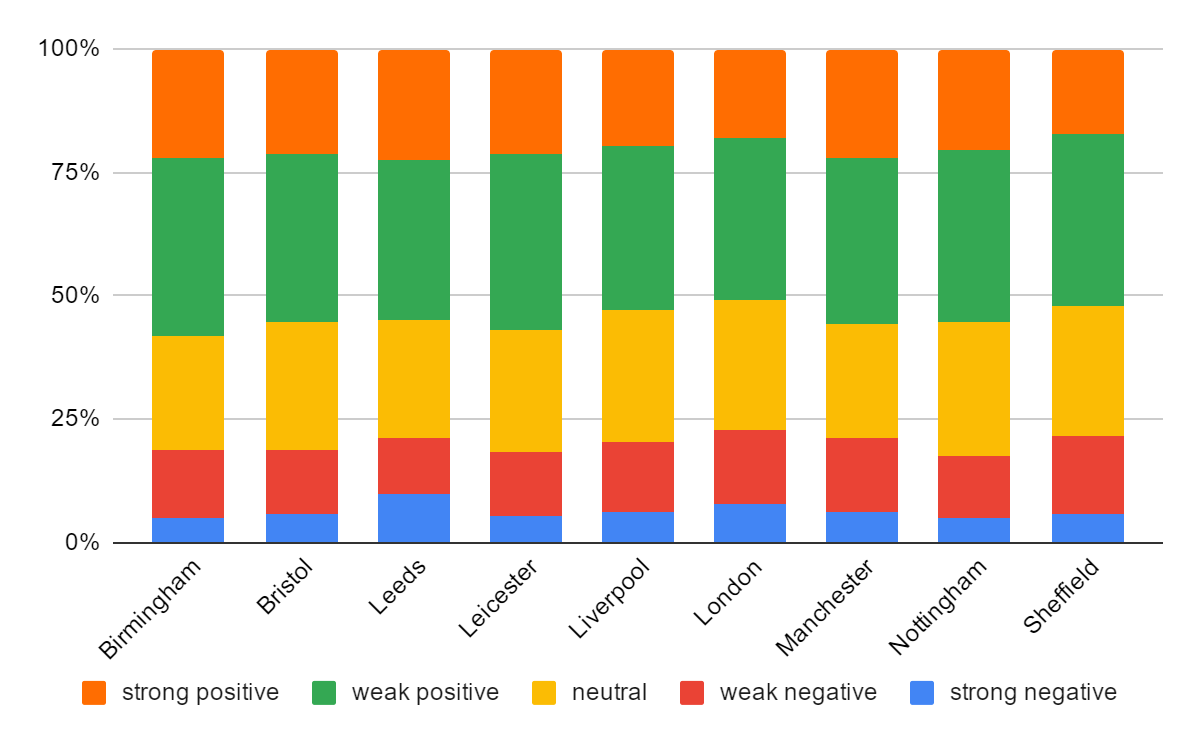
The distribution of sentiment expressions across a sample of UK cities.

In Figure 3, we present the proportions of five sentiments (strong positive, weak positive, neutral, weak negative, and strong negative) in the top 9 cities in our data in terms of their number of tweets. We can observe that approximately 60% of tweets were positive and 20% were negative in each city. At the same time, the percentage of tweets with different sentiments differed among these cities. For example, Leeds had a relatively high proportion of strong negative tweets and Sheffield had a relatively low proportion of strong positive tweets.

**Figure 4:**
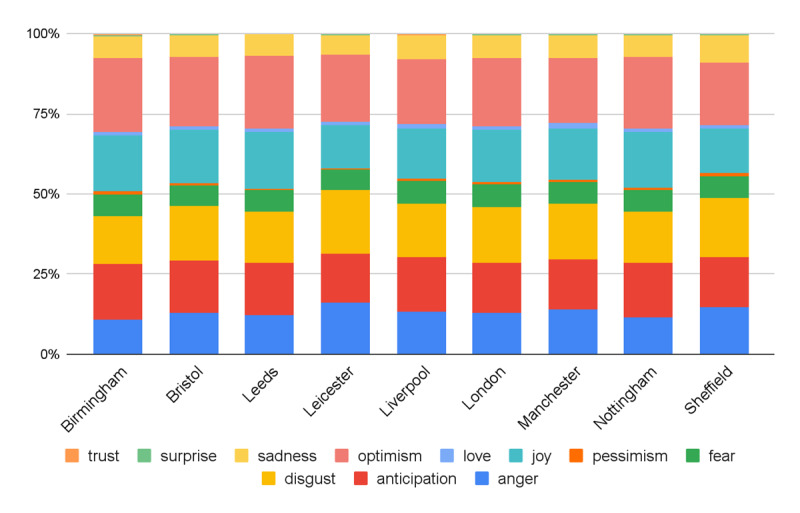
The distribution of emotion expressions across a sample of UK cities.

In Figure 4, we present the distribution of emotion expressions across the top 9 cities in our data. It can be observed that these 9 cities shared quite similar distributions, although the proportion differed from emotion to emotion. For instance, “optimism” and “anticipation” were the most frequently expressed emotions. We also noted some mixed emotions such as joy, disgust, and anger, which are reasonable feelings to be expressed during the COVID-19 pandemic. Interestingly, the proportion of trust expressions was extremely low, which could be linked to the lack of trust in decision-makers to deal properly with the situation due to the high infection rates. It is noteworthy that the proportion of trust expressions has been found to be generally scarce on Twitter in previous work [50,51].

**Figure 5:**
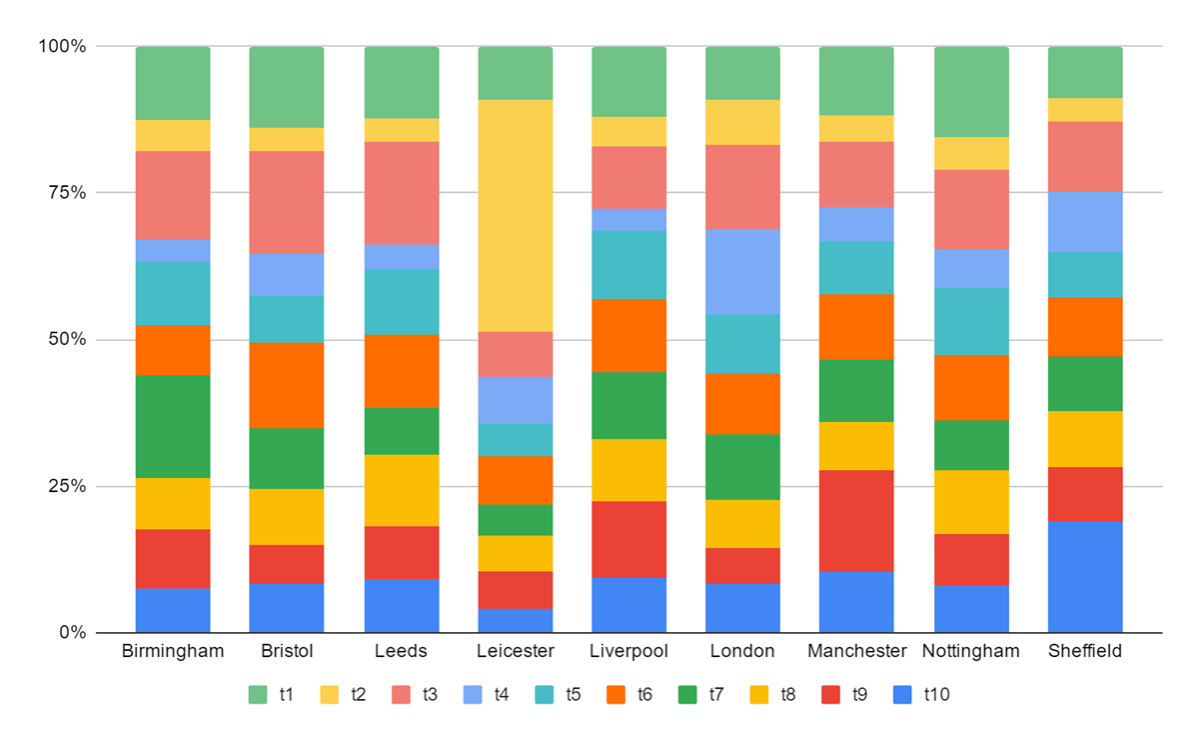
The distribution of topic expressions across a sample of UK cities. See Table 3 for a description of topics t1-t10.

In addition, we also counted the proportion of 10 topics in different cities, as shown in Figure 5. Similar topics received different degrees of attention in different cities. For instance, the main topic discussed in Leicester was t2 (*trade, worldwide*), which revealed that the public is more concerned about international trade. In London, the residents talked more about t4 (*kingdom, united*) than in other cities. In addition, Sheffield’s population focused more on the death topic given the higher proportion of t10 (*die, killing*) than found in the other cities.

### Analysis of Time

**Figure 6:**
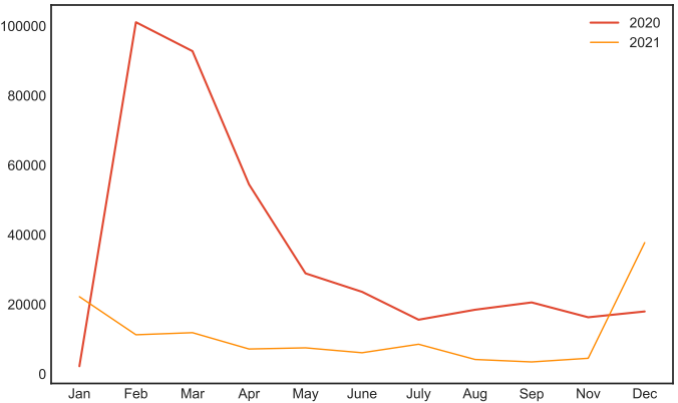
Number of tweets related to COVID-19 from January 2020 to December 2021. Each colored line represents a specific year (ie, red represents 2020, while orange represents 2021).

With time, the situation of the epidemic has changed, reflecting the level of concern about the epidemic. Figure 6 displays the number of tweets related to COVID-19 from January 2020 to December 2021. We can observe a sharp increase in the number of tweets from January 2020 to February 2020 (approximately 100,000 tweets), mainly due to the outbreak of COVID-19 in the United Kingdom. There was a gradual decline in the number of tweets as of February 2020, suggesting that people became less concerned about the epidemic. Moreover, the overall number of tweets was relatively low in 2021. With identification of the COVID-19 Omicron variant in the United Kingdom in November 2021, the number of tweets posted increased.

**Figure 7:**
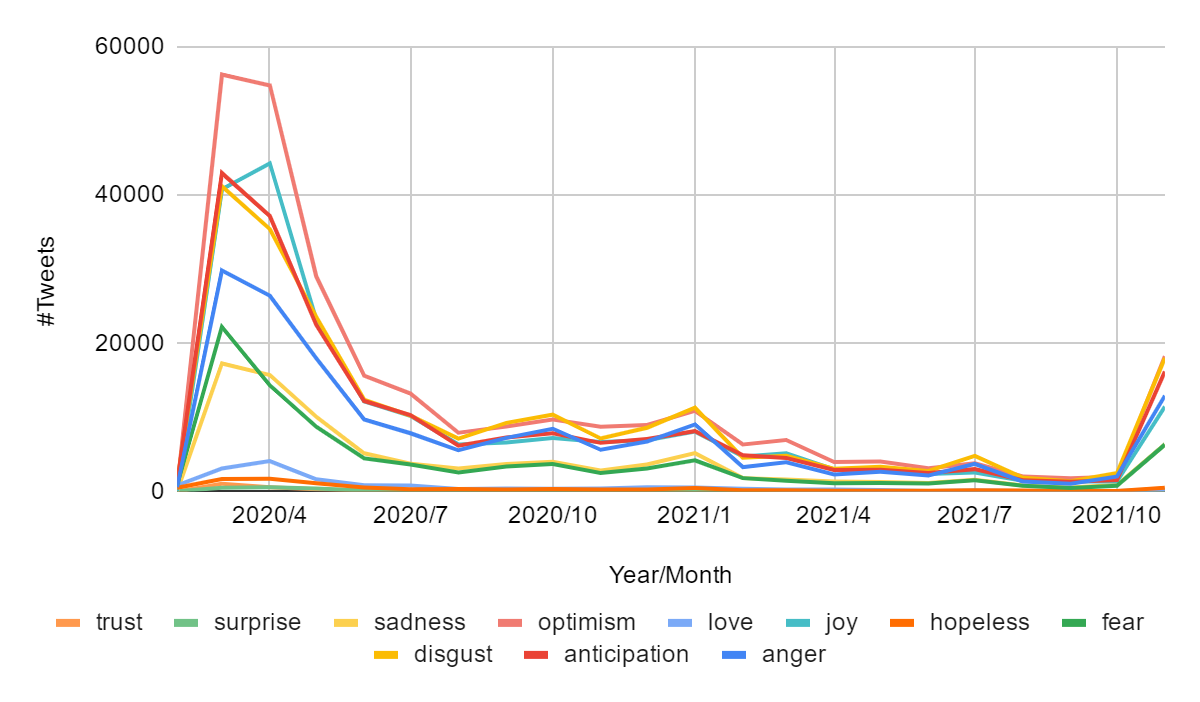
Number of tweets with different emotion expressions from 2020 to 2021.

Figure 7 presents the emotion expressions over time, covering the 2 years (ie, 2020 and 2021). We noticed that the distribution changed with time. In the beginning of 2020, almost all emotion labels displayed high peaks of expressions, with some obviously higher than others, such as optimism. As time progressed, the number of posted tweets containing emotions decreased, but the emotion distributions had dramatically changed from being highly positive to negative. This trend progressed until reaching the end of 2021. For instance, disgust, sadness, and hopelessness were among the top expressed emotions during this period, which were reasonable emotions to be expressed during this period since the number of cases and deaths increased [52].

**Figure 8:**
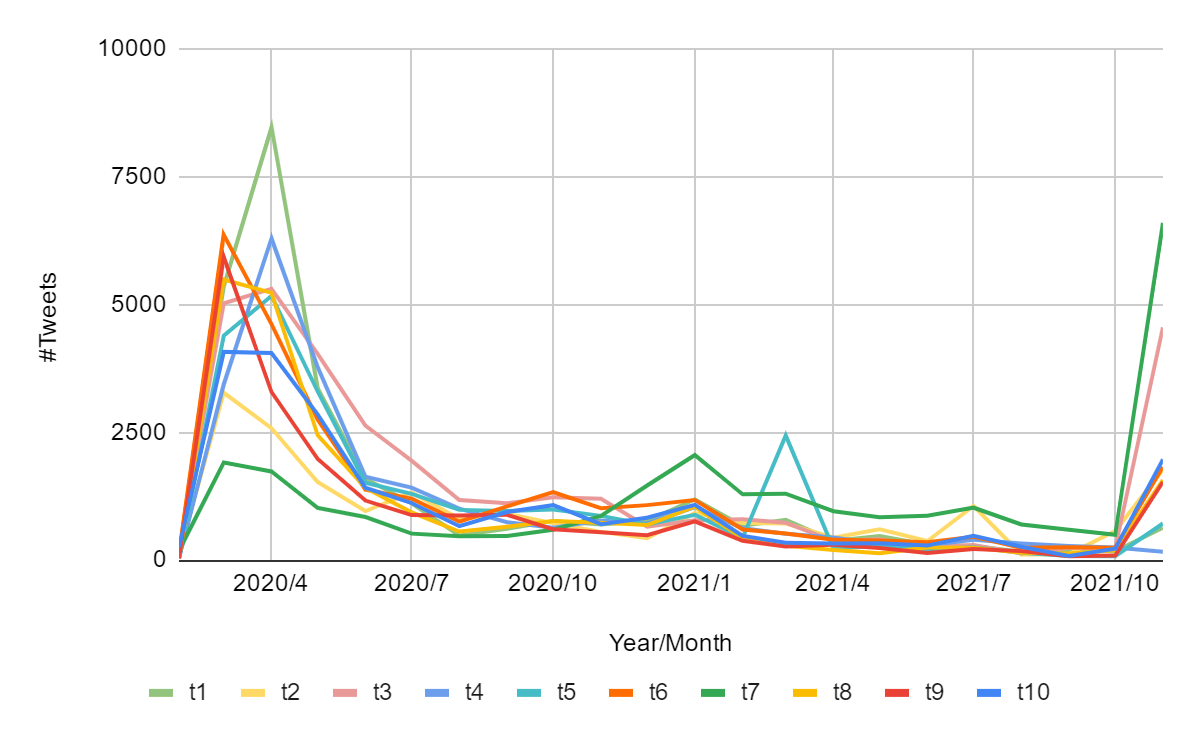
Number of tweets with different topic expressions from 2020 to 2021. See Table 1 for the descriptions of topics t1-t10.

Figure 8 shows the change in topics (among 10 selected topics) of all tweets between February 2020 and November 2021. We can see that the change is relatively significant. In April 2020, many tweets expressed gratitude to heroes of local councils for the epidemic, given the highest frequency of messages related to t1 (*grateful, thanks*). In addition, due to advances in vaccine research and an increase in the number of people vaccinated, the number of tweets referring to t7 (*vaccination*) relatively increased, and reached the highest value in January 2021. Interestingly, there were many tweets related to t5 (*film, videos*) because of the emergence of films with special significance, such as *A Beacon of Hope: The UK Vaccine Story* and *One Year On: A pandemic poem for Londoners*. For example, someone posted “What an honour to be filmed by @BBCLondonNews reading this part of our One Year On, poem marking the anniversary of the 1st lockdown.”

### Analysis of Examples

Multimedia Appendix 2 presents 9 instances from our data, each of which is linked to different attributes (ie, emotions, emojis, sentiment, and topics), demonstrating interesting findings that highlight the benefits of these attributes to the understanding of people’s reactions with respect to the pandemic. Here, we describe some use cases of emojis in tweets that were commonly observed across our data. Examples 1 and 3 display the use of emojis that are related to vaccine-taking (syringe emoji) and feeling strong/protected (muscle emoji). These two examples suggest that being vaccinated can make people feel strong and protected against the COVID-19 disease. Other examples (ie, Examples 4 and 5) discuss flight cancellation (airplane emoji), causing people to miss their already planned trips and holidays. Example 5 also discusses the potential of being able to travel again once the COVID-19 vaccine has been taken. A further example illustrates the benefits of developing volunteering programs that can assist hospitals and communities in fighting the COVID-19 crisis. Furthermore, the mask emoji was used in different ways, depending on the context (eg, lockdown for a long period).

From the perspective of sentiment, different tweets expressed different sentiments (including positive, neutral, and negative sentiments). Example 3 discusses that the second COVID-19 vaccine had been successfully administered and Example 9 praises community groups for their help and support, both of which show strong positive sentiment from the users. Example 7 expresses negative sentiment since the user could not see her relatives due to the epidemic. Some other examples (ie, Examples 1, 2, 5, 6, and 8) generally express positive attitudes during the pandemic by introducing vaccination, lockdown, or volunteers. In addition, Example 4 shows an instance that expresses mixed sentiment (positive and negative), although it was labeled by SenticNet as neutral. However, SpanEmo identified some mixed emotions, which helps to overcome the limitation of SenticNet in dealing properly with expressions having mixed sentiments or emotions.

Multimedia Appendix 2 also shows the top 3 topics for each example according to the probability calculated based on CTM. Examples 1, 3, and 5 belong to t7, which dominates the discussion of vaccination and boosters. Examples 4, 7, and 8 express the users’ attitudes and moods toward the impact of COVID-19 on their lives, and thus all of these were classified as t18. Examples 3 and 6 also belong to t1 (related to gratitude) because of the appearance of “thank you.” Moreover, discussion or usage of social media (t3) was expressed in some tweets (eg, Example 9).

## Discussion

### Principal Findings

This study explored more than 500,000 tweets related to COVID-19 between January 2020 and December 2021 in different cities of the United Kingdom, where the number of tweets increased dramatically following the outbreak. We used three deep learning–based models to analyze and combine sentiments, emotions, and topics to identify the key public concerns during the pandemic. Through our analysis, we found that emotion analysis can support understanding of people’s opinions and attitudes during the COVID-19 pandemic. Meanwhile, taking geolocation information into account can reveal differences between different areas in the United Kingdom. The overall sentiment was positive over time, and optimism was the predominant emotion, suggesting that people tend to be optimistic about the situation. There were changes in the sentiments, emotions, and topics expressed on Twitter as the epidemiological situation and government policies changed (eg, vaccination, social distancing) over these 2 years, which also reflect changes in people’s attitudes.

The benefits of the selected attributes for gathering evidence about people’s reactions during the pandemic in the United Kingdom were also identified. These attributes include emotion, sentiment, emojis, and topic modeling. This analysis demonstrated that such attributes can help gather evidence and analyze interactions between people during the pandemic. The first attribute was emotion, which can serve as a guide in understanding people’s reactions. For example, some people express concerns about COVID-19 for multiple reasons such as (1) taking a longer time to be resolved than expected, (2) cancelling or changing plans, (3) traveling restrictions, (4) wearing masks, and (5) isolation and lack of contact from family and friends. Others express some positive reactions and potential solutions for dealing with the pandemic, including family support, being inoculated with vaccines, staying at home or wearing masks, and volunteering. The second attribute was emojis, which describe the overall expression in the text, similar to topic modeling in the sense that both refer to the topics expressed in tweets. This provides another dimensionality for emojis, which have been used as a surrogate to collect emotion data [53,54]. Although this point is interesting to observe through this work, we leave it for future work to be investigated in greater depth.

Sentiment analysis is also useful to gain insight into the public opinion and perception behind certain events. By analyzing the sentiments in our data, we found that most people have had a positive attitude during the pandemic, which matches the conclusion of previous research [55], since they often post information related to good policies such as social support and vaccination to boost confidence in the fight against COVID-19. Definitely, some people still expressed worry about the outbreak and developed negative feelings due to the deaths, isolation, and lockdown policies, which affected their normal lives.

From the topics extracted, we found that there are many distinct topics people focus on, including symptoms of COVID-19, vaccination, social media, government policies, and living conditions. The changing themes of social media reveal the impact of COVID-19 on people’s lives, shifting the discussion about daily life to the pandemic and policies.

In addition, the emojis used, the emotions expressed, and the topics discussed by people who are from different cities in the United Kingdom all differed because of various factors such as the environment in the city, the epidemic situation, policies, and hot spots. The findings reveal the complexity and diversity of people’s perceptions toward the COVID-19 pandemic, which indicates the need to keep track of public attitudes.

### Limitations

This work is based on existing natural language processing methods that were used to analyze different attributes such as emotions, sentiment, and topics. However, these existing methods may not guarantee that their predictions reflect the actual attribute. In addition, emotion and sentiment are subjective tasks, which make them difficult to model and in turn could affect our interpretation as well as our results. Moreover, since our data were collected from Twitter with the use of specific keywords, it is possible that we missed other topics in online threads and viewpoints. Related discussions could also be taken from other social media platforms (eg, Facebook, Reddit). In this respect, our data provide a partial sample of user interactions on Twitter. The methods nevertheless are applicable to other longitudinal data and social media platforms.

### Conclusion

Our main contribution is the multimethod approach that provides insights into public sentiment and emotions in UK cities during the COVID-19 pandemic. Furthermore, our methods are location- and time-based, supporting a comparative analysis to track public concerns. Our analysis demonstrated that positive attitudes were common during the pandemic; optimism and anticipation were the dominant emotions. With the outbreak and epidemiological change, the government developed control measures and vaccination policies, and the topics also shifted over time. In addition, the comparative geolocation analysis revealed differences in the emotions expressed and topics discussed by people in different cities. Overall, our study shows that analyzing the data from social media can help to better understand public emotions and concerns related to COVID-19 at the city level, which will potentially enable developing acceptable policies.

## Appendix

Multimedia Appendix 1 Percentages of tweets in the data set associated with each individual emoji.

Multimedia Appendix 2 Examples of tweets expressing positive and negative reactions about COVID-19.

## Abbreviations

CTM: combined topic modeling
NHS: National Health Service
