# Supplementary material for: Emotions and Topics Expressed on Twitter During the COVID-19 Pandemic in the United Kingdom: Comparative Geolocation and Text Mining Analysis
Source: J Med Internet Res. 2022 Oct 5;24(10):e40323. doi: 10.2196/40323 (PMC9536769; doi:10.2196/40323)
Supplement: Multimedia Appendix 1 [file jmir_v24i10e40323_app1.pdf]

Percentages of tweets in the data set associated with each individual emoji. For completeness, a descriptive text of each emoji is also provided, which reveals the meaning of each emoji.

🙏	👇	🦠	👏	😂	GB	😷	❤️	👍	🤔
hands pressed together	pointing down	virus	clapping hands	face with tears of joy	United Kingdom	face with medical mask	red heart	thumbs up	thinking face
5.48	3.16	2.96	2.73	2.62	2.45	2.33	2.24	2.02	1.97
💙	💉	♂️	🤣	💪	😊	🤦‍♀️	👉	🙄	♀️
blue heart	syringe	man symbol	rolling on the floor laughing face	feats of strength	smiley face	facepalm	pointing right	eye roll	woman symbol
1.44	1.29	1.2	1.19	1.16	1.09	1.05	1.04	1.03	0.99
🌈	🙌	♥️	🤷‍♀️	🍺	😡	✅	😢	⬇️	😭
rainbow	hands raised in celebration	hearts suit	shrug	beer mug	mad face	check mark symbol	crying face	down arrow	loudly crying face
0.99	0.95	0.95	0.91	0.86	0.85	0.84	0.65	0.6	0.59
😁	➡️	😳	🌍	🤬	😔	👀	👌	💚	🥰
grinning face	right arrow	face with wide open eyes	planet	face with symbols over mouth	sad pensive face	eyes	oK hand sign	green heart	smiling face with hearts
0.58	0.58	0.57	0.56	0.55	0.52	0.51	0.51	0.51	0.48
💀	😍	🚨	💔	😉	😎	💜	😅	🤞	✊
skull	smiling face with heart-eyes	police car's light	broken heart	winking face	smiling face with sunglasses	purple heart	grinning face with sweat	fingers crossed	raised fist
0.46	0.46	0.45	0.42	0.4	0.4	0.38	0.37	0.35	0.35
