# Supplementary material for: Emotions and Topics Expressed on Twitter During the COVID-19 Pandemic in the United Kingdom: Comparative Geolocation and Text Mining Analysis
Source: J Med Internet Res. 2022 Oct 5;24(10):e40323. doi: 10.2196/40323 (PMC9536769; doi:10.2196/40323)
Supplement: Multimedia Appendix 2 [file jmir_v24i10e40323_app2.pdf]

Examples of tweets expressing positive and negative reactions about COVID-19. The columns from left to right correspond to the tweet, emoji, sentiment (sent), emotion (emo), and topics, respectively. See Table 3 for a description of each topic.

Tweet	Emoji	Sent	Emo	topics
1. I finally had my first #COVID19 vaccination jab today. Now I've gotta wait 6 weeks till I have my second. #GetVaccinated 💉💪💯	💉💪 💯	weak pos	anti joy optimism	t7 t17 t6
2. One year on...what a prolific year it's been. Hard to comprehend at times. Never thought we'd still be in lockdown 12 months on 😷😷😷#DayOfReflection2021 #Covid19 #OneYearOn #nationaldayofreflection	😷	weak pos	anger disgust fear sadness	t17 t13 t16
3. So happy to have 2nd #COVID19 jab tonight! Thank you #TraffordGeneral @NHSEngland - I feel so much safer now!💉🦠😷	💉🦠😷	strong pos	joy love optimism	t1 t7 t16
4. No more head down....head up. 👀 I feel COVID19 🦠 is closing in. One of my races has just been cancelled - @flingrace I respect their responsible decision despite loss of ✈️/ logistics £☹️. How are things looking for you? Priority - Stay #healthy #positivityRun 🏃 Run	👀 🦠 ☹️ ✈️	neutral	optimism sadness	t18 t17 t9
5. My first #COVID19 vaccination done. Painless and quick,That's one step closer to travelling again✈️🌍 #gemstonedetective 🥳💪🏽	💪 🥳 🌍 ✈️	weak pos	anticipation joy optimism	t6 t7 t17
6. We are deploying @stjohnambulance volunteers on ambulances 🚑, in hospitals 🏥, and in the community💚.#ThankYou to Aidan and others for all they have done, and will do, during the #COVID19 crisis. #VolunteersWeek2020	💚 🚑 🏥	weak pos	joy optimism	t1 t11 t14
7. Crying my eyes out because this #MothersDay sucks 😥 I can't see my beautiful daughter and I can't see my mum I hate this 😭 #COVID19 #MothersDay	😥😭	weak neg	anger disgust sadness	t18 t17 t14
8. "If anything, this #coronavirus #lockdown, is a testament to true human creativity! Look at these guys 😂❤️ Keep the positivity up everyone. Hopefully, with a smile! #COVID19	😂❤️	weak pos	anticipation joy optimism	t18 t6 t13
9. Delighted to see #covid19 Mutual Aid neighborhood groups are springing up across	🙌	strong pos	joy optimism	t3 t17 t1

London. This is when social media can be constructive. Helping others can be a great form of #selfcare during #covid19UK 🙌				
